# Sodium dichloroisocyanurate delays ripening and senescence of banana fruit during storage

**DOI:** 10.1186/s13065-018-0503-5

**Published:** 2018-12-05

**Authors:** Qixian Wu, Taotao Li, Xi Chen, Lingrong Wen, Ze Yun, Yueming Jiang

**Affiliations:** 1 Key Laboratory of Plant Resources Conservation and Sustainable Utilization, Guangdong Provincial Key Laboratory of Applied Botany, South China Botanical Garden, Chinese Academy of Sciences, Guangzhou, 510650 People’s Republic of China; 2 University of Chinese Academy of Sciences, Beijing, 100039 People’s Republic of China

**Keywords:** Banana fruit, Sodium dichloroisocyanurate, Ripening, Ethylene, Antioxidant activity, Metabolomics

## Abstract

Banana as a typical climacteric fruit soften rapidly, resulting in a very short shelf life after harvest. Sodium dichloroisocyanurate (NaDCC) is reported to be an effectively antibacterial compound. Here, we investigated the effects of NaDCC on ripening and senescence of harvested banana fruit at physiological and molecular levels. Application of 200 mg L^−1^ NaDCC solution effectively inhibited the ripening and senescence of banana fruit after harvest. NaDCC treatment reduced greatly ethylene production rate and expressions of genes encoding *1*-*aminocyclopropane*-*1*-*carboxylate synthetase*, *1*-*aminocyclopropane*-*1*-*carboxylate oxidase*, *ethylene*-*responsive transcription factor* and *EIN3*-*binding F*-*box protein*. Meanwhile, NaDCC treatment down-regulated markedly the expressions of *xyloglucan endotransglucosylase/hydrolase* and *pectinesterase* genes. Furthermore, NaDCC treatment affected significantly the accumulation of ripening-related primary metabolites such as sugars and organic acids. Additionally, NaDCC treatment decreased the production of hydroxyl radical and increased 1,1-diphenyl-2-picrylhydrazyl (DPPH) scavenging activity, reducing power and hydroxyl radical scavenging activity. In conclusion, NaDCC delayed effectively the ripening and senescence of harvested banana fruit via the reduced ethylene effect and enhanced antioxidant activity.

**Electronic supplementary material:**

The online version of this article (10.1186/s13065-018-0503-5) contains supplementary material, which is available to authorized users.

## Introduction

Banana (*Musa* spp., AAA group, cv. ‘Brazil’) as a major fruit in tropical and subtropical area is consumed around worldwide because of its high production [1]. As a climacteric fruit, banana fruit requires ethylene effect for ripening [2], which results in a rapid softening progress [3]. Along with fruit senescence, peel spotting and fungous infection appear easily on the fruit surface [4]. Thus, quality deterioration induced by these above-mentioned factors results in a very short shelf life of banana fruit after harvest, which causes great financial loss. It is required urgently to develop effective postharvest technologies and facilities to maintain the sensory quality and extend the shelf life of harvested banana fruit during marketing.

For climacteric fruit such as banana, ethylene induces fruit ripening [5]. The 1-aminocyclopropane-1-carboxylate synthase (ACS) and 1-aminocyclopropane-1-carboxylate oxidase (ACO) are related to the sharp ethylene production in climacteric fruit, which initiates the changes in color, texture, aroma and flavor and other physiological attributes [6]. Cheng et al. [7] reported that nitric oxide (NO) treatment can reduce greatly production of ethylene which was associated with low expression of *MA*-*ACS1* and *MA*-*ACO1* genes in banana fruit. Meanwhile, 1-pentylcyclopropene (1-PentCP), a potential ethylene inhibitor, delayed markedly the change in skin color and inhibited the activities of ACS and ACO which were associated with the suppressed gene expressions of ethylene response sensor 1 (*MA*-*ERS1*) and ethylene-responsive transcription factor 1 (*MA*-*ERF1*) of banana fruit [8]. Moreover, *EIN3* binding F-box proteins (*EBFs*) were shown to regulate *EIN3/EIL* turnover in ethylene signaling pathway. For example, *MaEBF1* plays an important role in the initial phase of ethylene signaling [9]. Additionally, the regulation of *DkERFs* bound directly to the *DkXTH9* promoter affected fruit softening of persimmon fruit [10]. Thus, the regulation of ethylene synthesis depends largely on fruit ripening and senescence and shelf life of harvested banana fruit.

The imbalance of reactive oxygen species (ROS) is also related to fruit abnormal ripening. For example, hydroxyl radical (·OH) can cause oxidation injury which leads to the cell wall disassembly and quality deterioration of banana fruit during storage [11]. Ren et al. [12] suggested that the improving quality and prolonging shelf life of mango fruits can be achieved by reducing oxidative damage caused by ROS during ripening. Huang et al. [13] reported that oxalic acid treatment could delay banana fruit ripening and inhibit the oxidative injury caused by excessive ROS. Recent research shows that reactive oxygen and nitrogen species (ROS/RNS) are involved in fruit ripening, during which molecules, such as hydrogen peroxide (H_2_O_2_), NADPH, nitric oxide (NO), peroxynitrite (ONOO–), and *S*-nitrosothiols (SNOs), interact to regulate protein functions through post-translational modifications [14]. ROS metabolism can depend on ethylene action also [15] and, thus, influences ripening and senescence and shelf life of banana fruit.

Metabolite is another important factor to indicate fruit ripening and senescence. A characteristic change in metabolite profile occurs during fruit ripening [16–18]. Nieman et al. reported that fructose concentration increased during banana fruit ripening [17] while the profile of soluble metabolites exhibited complex accumulation patterns (some are upregulated and some are downregulated) during kiwifruit ripening [18]. Metabolomics can provide comprehensive qualitative and quantitative description of metabolites and then can help to understand better the mechanism of fruit quality during ripening and senescence.

**Fig. 1:**
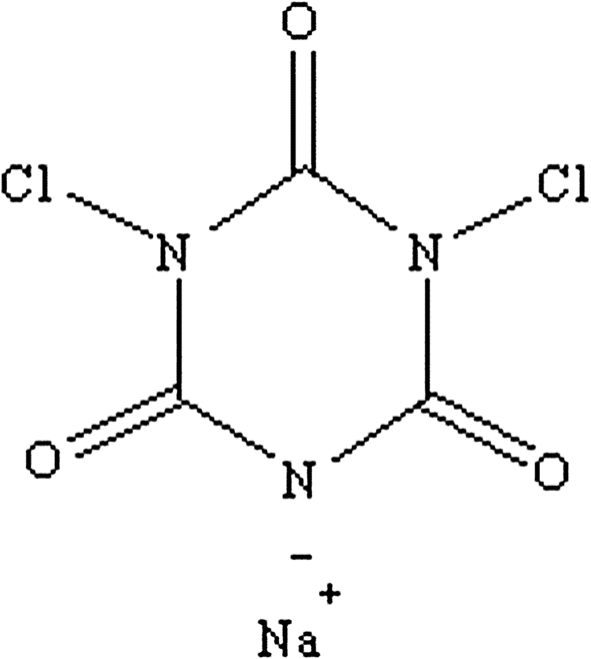
The structural formula of NaDCC (stored at room temperature)

Sodium dichloroisocyanurate (NaDCC) is reported to have great efficacy in killing microorganisms present in water, environmental surface and medical equipment [19]. NaDCC consists of two reactive chlorine atoms (Fig. 1) and can damage cell membranes, nucleic acid and proteins resulting in oxidative degradation of microorganism [20]. It is reported for instance that NaDCC can kill *Escherichia coli*, *Staphylococcus aureus*, *Debaryomyces hansenii*, *Aspergillus brasiliensis*, *Entamoeba histolytica*, *Giardia lamblia*, *Cryptosporidium*, *Cyclospora* and *Microsporidia* [19, 21]. NaDCC is widely used as a safe disinfection tablet in daily life and industries. Previous research indicated that application of NaDCC at 50 ppm can prolong shelf life of fresh-cut onion with higher pH value and lower titratable acidity [22]. Additionally, NaDCC treatment in combination with gamma irradiation is able to control soft rot disease caused by *Rhizopus* of sweet potatoes, pears and paprikas after harvest [23–25]. Thus, NaDCC shows the potential for application for improving quality and prolonging shelf life of postharvest fruits because of its antibacterial and properties.

The objective of this present study was to investigate the effect of NaDCC on the ripening and senescence of banana fruit during storage. The integrative analyses of physiological parameters, profile of primary metabolites and gene expression were conducted to obtain insight in the molecular and metabolic effects of NaDCC treatment on fruit ripening and senescence caused by NaDCC treatment. This study will be beneficial to develop new postharvest technology to maintain quality and extend shelf life of banana fruit.

## Results and discussion

### Effect of NaDCC treatment on fruit ripening and senescence

**Fig. 2:**
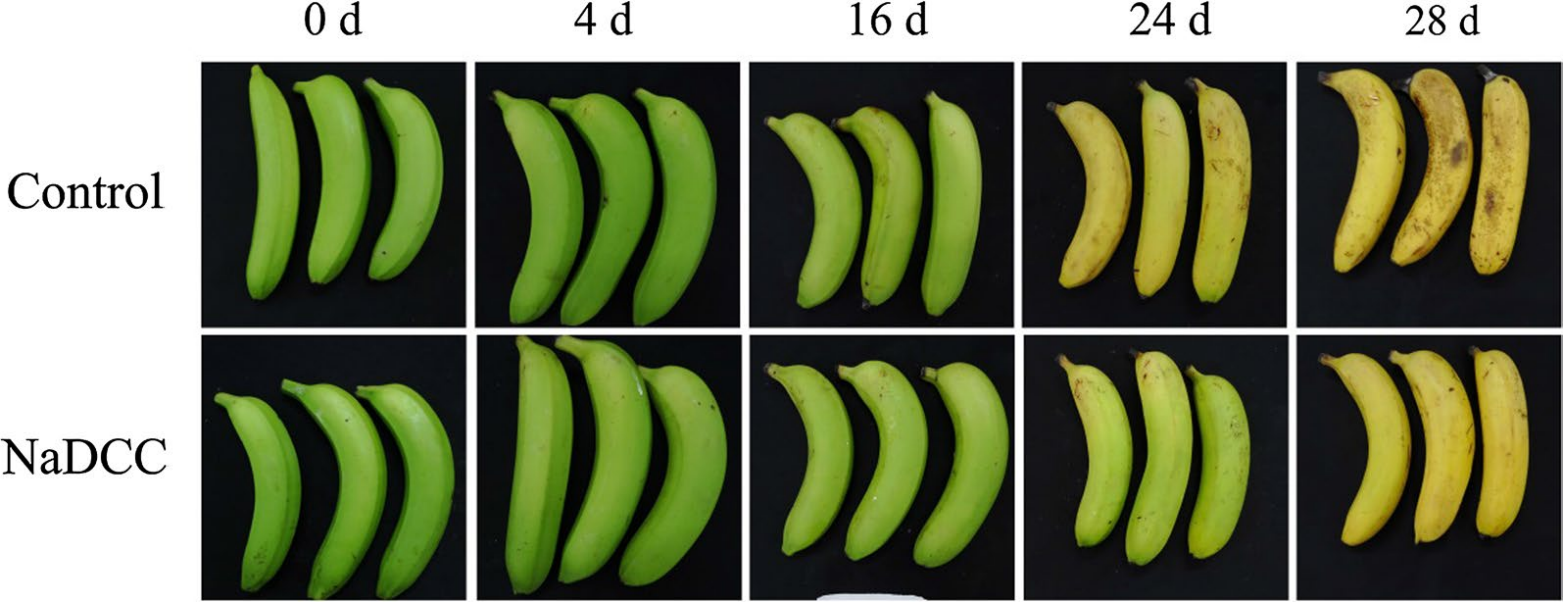
Changes in visual appearance of the banana fruit during storage

**Fig. 3:**
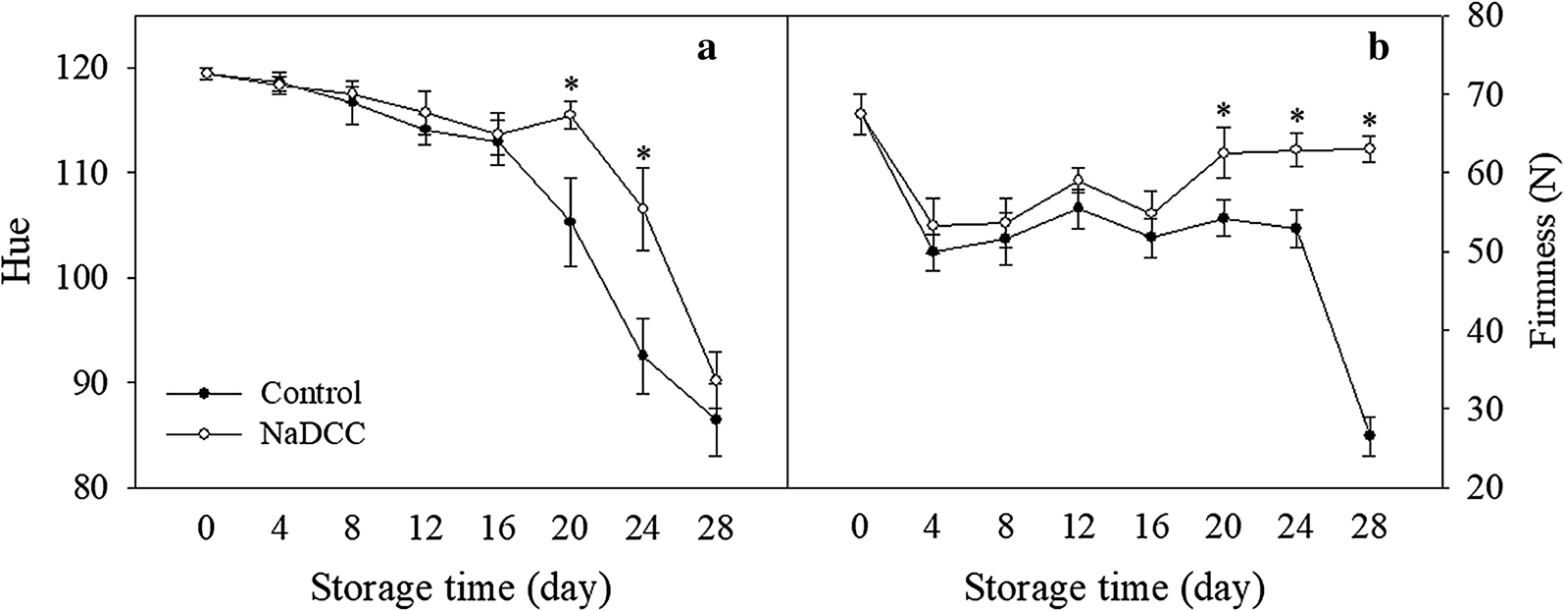
Changes in hue angle (**a**) and fruit firmness (**b**) of banana fruit during storage. Data presented are means (from three separate groups) ± standard errors (n = 3). The asterisks above bars represent a significant difference (*p *< 0.05)

**Fig. 4:**
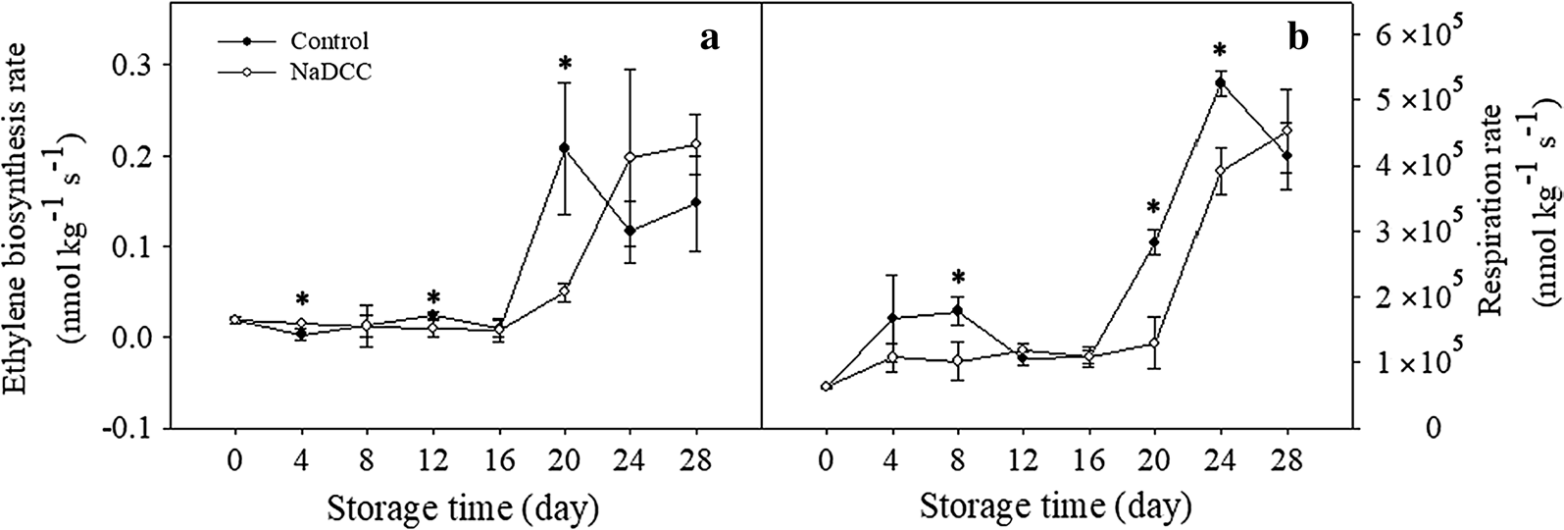
Changes of ethylene biosynthesis rate (**a**) and respiration rate (**b**) of the stored banana fruit. Data presented are means (from three separate groups) ± standard errors (n = 3). The asterisks above bars represent a significant difference (*p *< 0.05)

Green mature banana fruit turns gradually into yellow. In this study, NaDCC treatment could significantly delay the ripening process of banana fruit (Fig. 2). The color chroma indexes for control and NaDCC-treated fruit diminished gradually during storage but the control fruit decreased more markedly (Fig. 3a). In contrast to color, the changes in firmness of the NaDCC-treated fruit were slower than control fruit (Fig. 3b). Especially after NaDCC treatment, the fruit firmness after 28 days of storage was 63 N, which was higher than the control fruit (26.50 N) (Fig. 3b). Previous study reported that NaDCC was used as a chloric antibacterial agent (Fig. 1) in raw vegetables and fruits [21, 23, 25] and NaDCC was efficient in the control of antifungal infection of harvested guava and paprika [25, 26]. In this study, it was found that NaDCC showed a potential in delaying fruit ripening and maintaining firmness. Application of NaDCC delayed the appearance in the peaks of ethylene production (8 days) and respiration (4 days) rates (Fig. 4a, b). The increasing production of ethylene and carbon dioxide are important indicators of fruit ripening and senescence [27, 28]. Sodium hypochlorite treatment, a chloric antibacterial agent, decreased the respiration rate and ethylene production of tomato fruit [29]. Altogether, our results showed that NaDCC treatment can delay the ripening, softening and extend storage time of banana fruit through inhibition of CO_2_ and ethylene production rate during storage.

### Effect NaDCC treatment on related genes expression of ethylene synthesis and cell wall degradation

**Fig. 5:**
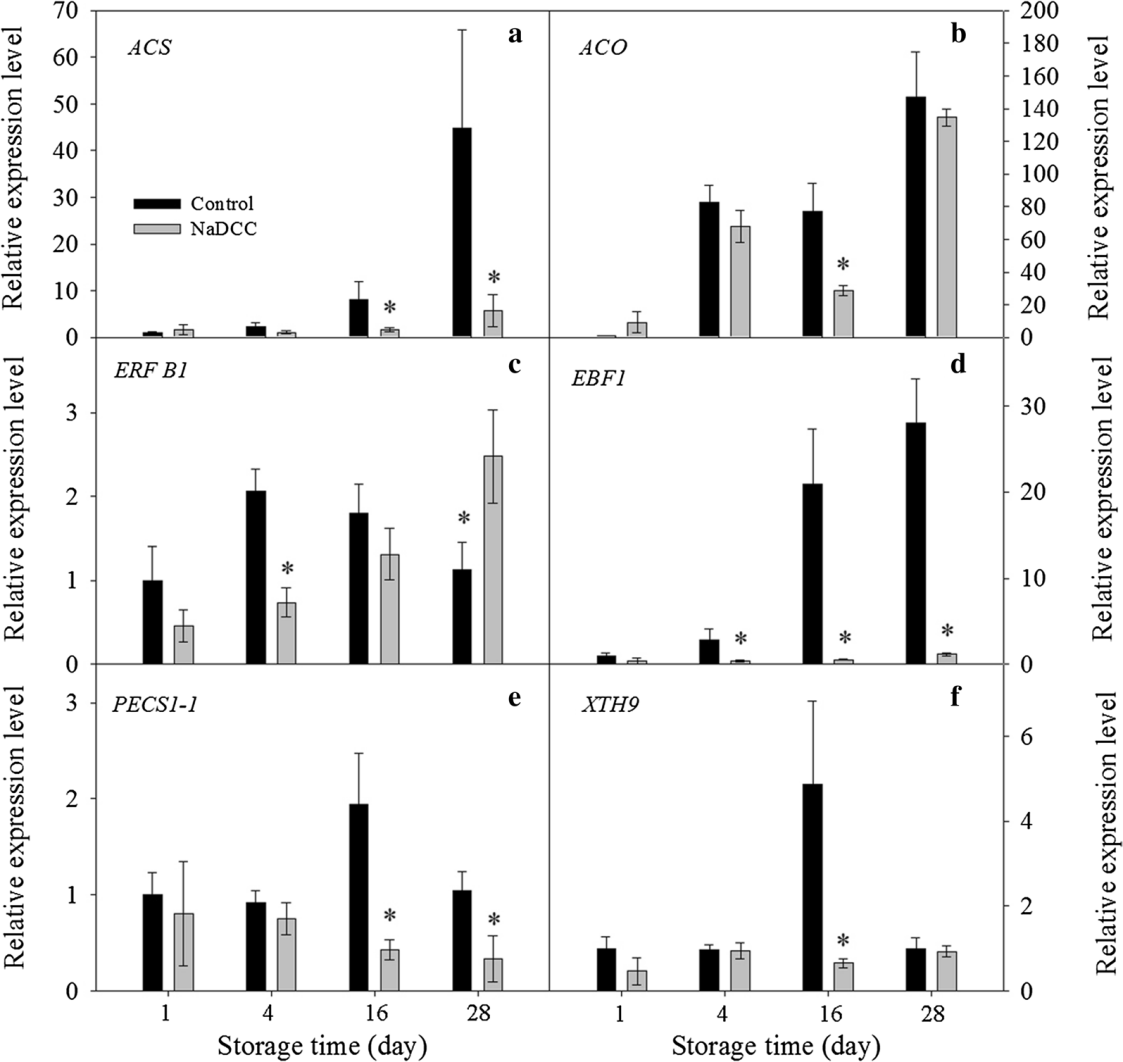
Changes in the relative expression levels of peel tissue of banana fruit during storage. Gene expression in Control fruit after 1 day of storage was set as 1. Each data point represents a mean (from three separate groups) ± standard errors (n = 3). The values with asterisks are significantly different (*p *< 0.05)

As mentioned above, ethylene plays a key role in fruit ripening and senescence. It is widely accepted that ACS and ACO are the main enzyme of ethylene biosynthesis pathway [30]. In order to understand the inhibitory effect of NaDCC treatment on fruit ripening and senescence, the expression of ethylene-biosynthesis was analyzed comparatively. NaDCC treatment decreased markedly the expression levels of *MaACS*, *MaACO*, *MaEBF1* and *MaERF1B*, which was in agreement with an inhibited tendency of ethylene production rate. In this study, the gene expression level of *MaACS* of banana fruit treated with NaDCC was reduced significantly at 16 days and 28 days of storage (Fig. 5a). As for *MaACO*, the gene expression level showed a decrease pattern during storage after NaDCC treatment (Fig. 5b). Except for the main enzyme of ethylene biosynthesis pathway, some ethylene-responsive factors play also vital roles in ethylene production during fruit ripening and senescence. The gene *ERFs* is the ethylene-responsive factors which comprise a large family of transcriptional factors [31]. In this study, the expression levels of two genes (*MaEBF1* and *MaERF1B*) were decreased markedly by the NaDCC treatment in an early storage period (Fig. 5c, d). Furthermore, as shown in Fig. 5, *MaEBF1* was affected more significantly compared with *MaERF1B*. Binder et al. [9] reported that *MaEBF1* plays a role in the initial phase of ethylene signaling. Hence, our study suggested that ethylene signaling was disturbed also by the inhibition of *ERF* and *EBF* gene expressions in the NaDCC-treated fruit.

Cell wall modification is related to fruit softening. Polygalacturonase (PG) and pectinesterase (PEC) are the major enzymes that can degrade synergistically the pectin in cell wall and PEC can degrade high methoxyl pectin into low methoxyl pectin further converted by polygalacturonase (PG) [32]. Furthermore, xyloglucan endotransglucosylase/hydrolase (XTH) can degrade xyloglucan and then affect the cell wall expansion [33]. The study showed that *MaPECS*-*1.1* gene expression decreased more markedly in the NaDCC-treated fruit compared with the control fruit at 16 days and 28 days of storage (Fig. 5e). Additionally, NaDCC treatment significantly inhibited the *MaXTH9* gene expression of banana fruit (Fig. 5f). This agrees with data reported by Mbéguiéambéguié [34] who that *MaPEs* and *MaXTHs* increased significantly during banana fruit ripening and senescence. Thus, the down-regulation of these two genes of the NaDCC-treated fruit was parallel with delayed decrease in firmness.

### Effect of NaDCC treatment on the radical scavenging activity

**Fig. 6:**
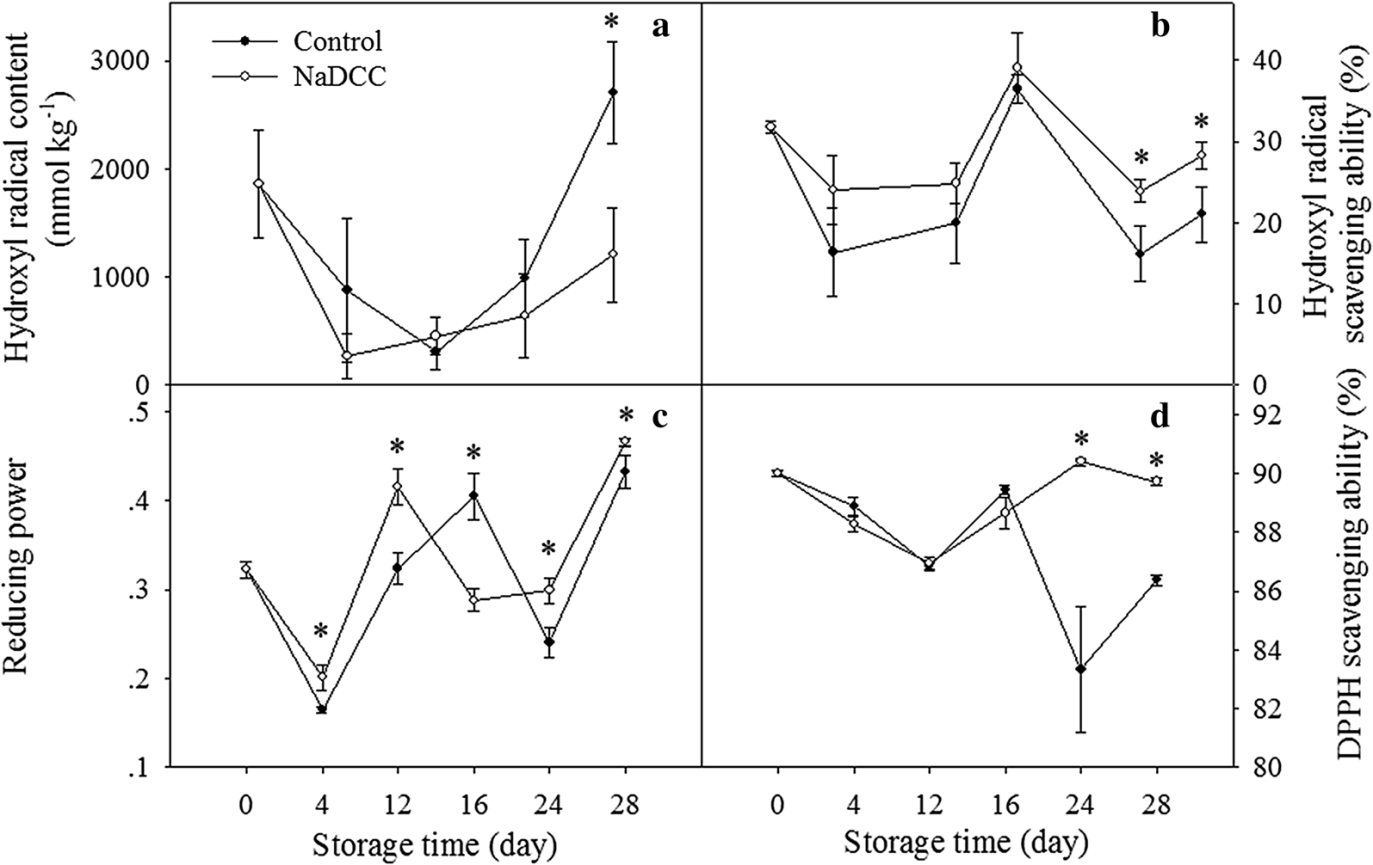
Changes in hydroxyl radical content (**a**), hydroxyl radical scavenging ability (**b**), reducing power (**c**) and DPPH scavenging ability (**d**) of banana fruit during storage. Each data point represents a mean (from three separate groups) ± standard errors (n = 3) and the values with asterisks are significantly different (*p *< 0.05)

Increasing accumulation of ROS can lead to the biological disorder [15]. Redox regulation is involved the imbalance between the production and scavenging of ROS during fruit ripening and senescence [35]. In this study, NaDCC treatment enhanced hydroxyl radical scavenging activity (Fig. 6a), which was in agreement with a lower content of hydroxyl radical in peel tissues (Fig. 6b). Furthermore, although NaDCC treatment didn’t affect the 1,1-diphenyl-2-picrylhydrazyl (DPPH) scavenging activity within the first 16 days of storage the NaDCC-treated fruit after 24 days showed a significant higher activity than control fruit (Fig. 6d). Additionally, a similar trend in reducing power was observed, and the NaDCC treatment advanced the peak value of the reducing power for 4 days (Fig. 6c). Strangely, the change of reducing power showed strong fluctuations. Considering reducing power serves as a vital indicator of antioxidant activity, the fluctuations of reducing power might be related to the complex change of antioxidants contents, composition, even the molecular weight during banana ripening process. However, the exact reason for the strong fluctuations of reducing power still need further research in future study. Though fluctuations of reducing power occurred during storage period, NaDCC treatment still significantly enhanced reducing power during banana fruit ripening period, except for 16 days. Previous study showed that the ROS balance affects greatly cell wall disassembly at various ripening stages of harvested banana fruit [11]. Considering the role of hydroxyl radical in modification of cell wall polysaccharides [36], we postulated that the inhibition of hydroxyl radical by NaDCC might also contribute to the firmness maintenance of banana fruit. As has been reported by Huang et al. [13] that oxalic acid could increase the radical scavenging capability to delay banana fruit ripening process. Excessive accumulation of ROS caused by down-regulation of antioxidant enzymes was related to the peel senescence during mandarin fruit storage [37]. Hence, the reduction of content of hydroxyl radical and the promotion of reducing power, hydroxyl radical and DPPH scavenging ability in NaDCC-treated fruits during storage might help to delay the softening process.

### Effect of NaDCC on the accumulation of many primary metabolites

**Fig. 7:**
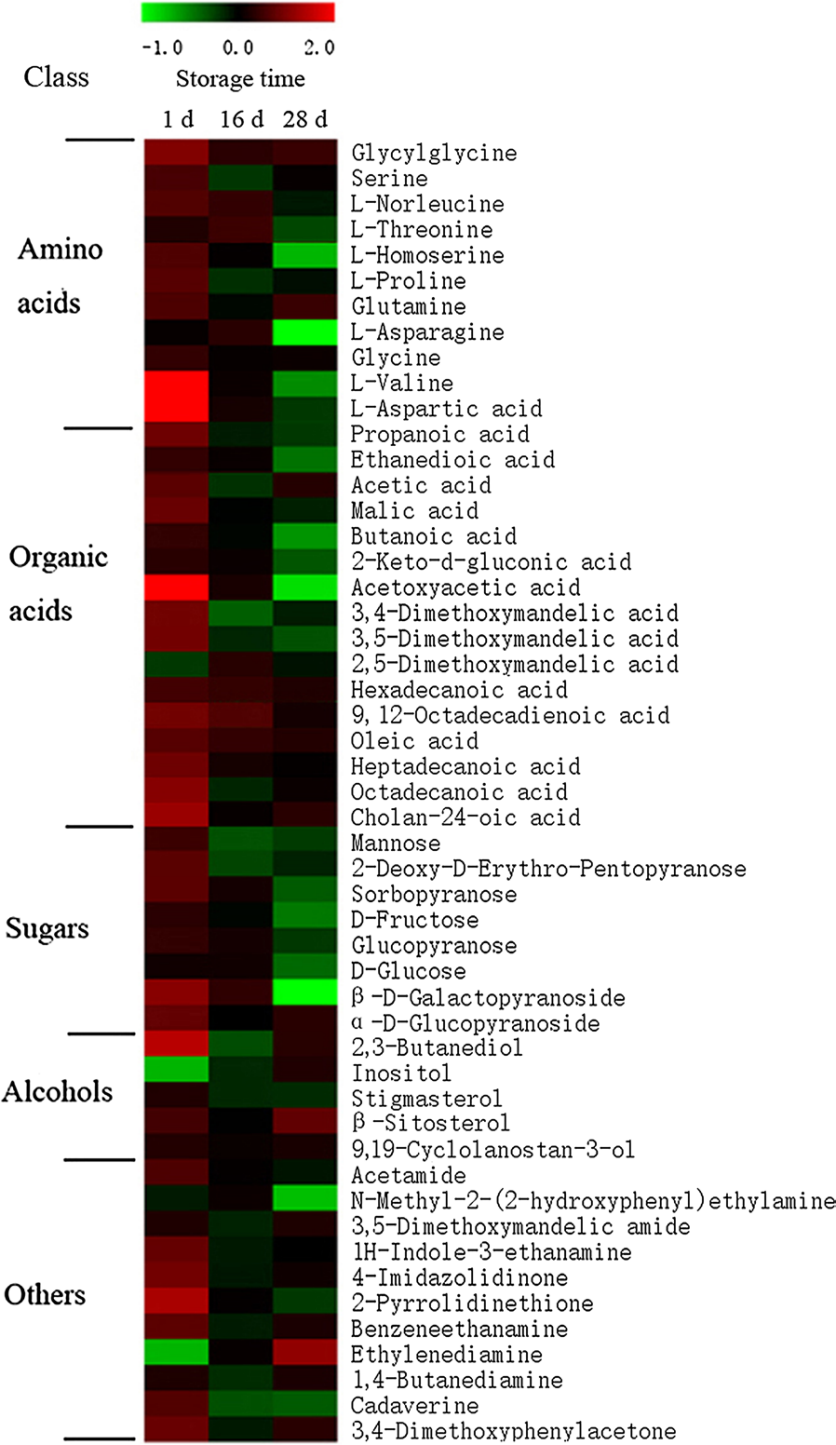
A profile of the primary metabolites of banana fruit during storage. Samples from Control and NaDCC-treated fruit after 1, 16 and 28 days of storage were used for profiling the primary metabolites. The primary metabolites were determined with GC–MS. Fifty-one metabolites increased/decreased markedly in the peel tissues of NaDCC-treated fruit compared with the control fruit. The scaling numbers (− 1, 0, + 2) stand for the log_2_ transformation values of the ratio between treated and control values

The primary metabolites are composed of sugars, amino acids, organic acids and alcohols. Changes in primary metabolites can use to help to understand fruit ripening [38, 39]. It was reported that the principal metabolites for ripening and senescence of harvested banana were determined to be valine, alanine, aspartic acid, choline, acetate, glucose, malic acid, gallic acid and dopamine [40]. In this study, GC–MS was used to measure the effect of NaDCC treatment on primary metabolites. Except for impurities, 52 metabolites were identified and selected in this study (Additional file 1: Figure S1 and Additional file 2: Figure S2). The detailed information of these metabolites is shown in Additional file 3: Table S1. Among these metabolites, 51 were significantly (*p *< 0.05) different between control and NaDCC-treated fruit, which mainly contained sugars, organic acids, alcohols, amino acids and other metabolites (Fig. 7).

Amino acids are vital nutrients in banana fruits [40]. Among these differentially accumulated metabolites, 11 amino acids showed markedly different accumulation patterns, except for l-alanine. The contents of serine, l-norleucine, l-threonine, l-homoserine, l-asparagine, l-valine, l-aspartic acid and l-proline decreased significantly during storage while glycine showed no differences between NaDCC-treated and control fruit after 16 days and 28 days of storage. The changes in these amino acids can influence nutritional and flavor quality of strawberry fruit [41]. During storage, the concentration of glutamine increased markedly. Glutamine showed a closely positive correlation with shelf life of tomato fruit [42], and, thus, the increase in glutamine concentration could be consider as a marker of long shelf life. Yuan et al. [40] found that valine and aspartic acid were characteristic marker of banana fruit senescence. In this study, down-regulation of l-valine and l-aspartic acid were associated with banana fruit ripening and senescence.

As for the major sugars, mannose, 2-deoxy-d-erythro-pentopyranose, sorbopyranose, d-fructose, glucopyranose, α-d-glucopyranoside and β-d-galactopyranoside increased within the 1st day. After 16 days of storage, NaDCC treatment increased sorbopyranose, glucopyranose, glucose and β-d-galactopyranoside (Fig. 7). It is noted that mannose and 2-deoxy-d-erythro-pentopyranose exhibited an opposite accumulation pattern compared with other sugars after of 16 days of storage, but at 28 days for β-d-galactopyranoside were inhibited markedly (Fig. 7). At the ripening stage, sugar accumulation can be observed by the degradation of starch into sucrose [43, 44]. The increase in d-fructose and d-glucose was beneficial for quality maintenance and storage extension [2]. As mannose has been identified in xyloglucan as a primary cell wall hemicellulose, the down-regulation of mannose after the NaDCC treatment may maintain hemicellulose [39], which can help to maintain firmness of banana fruit during storage. These results suggested that NaDCC treatment may strengthen cell wall maintenance.

Most organic acids increased at 1 day and 16 days, but decreased after 28 days of storage. As for climacteric fruits, previous studies indicated patterns in fatty acid composition in tomato [45], mango [46] and avocado [47] during fruit ripening. Deshpande et al. [46] found that saturated and unsaturated fatty acids increased significantly during mango ripening. NaDCC treatment of banana maintained high contents of 16 organic acids (Fig. 7). 15 of the organic acids were up-regulated significantly at 1 day except for 2,5-dimethoxymandelic acid. Furthermore, significantly increased contents of 2,5-dimethoxymandelic acid, hexadecanoic acid, 9,12-octadecadienoic acid, oleic acid and heptadecanoic acid but reduced contents of propanoic acid, acetic acid, 3,4-dimethoxymandelic acid, 3,5-dimethoxymandelic acid and octadecanoic acid for 16 days and decreased contents of propanoic acid, ethanedioic acid, malic acid, butanoic acid, 2-keto-d-gluconic acid, acetoxyacetic acid, 3,4-dimethoxymandelic acid and 3,5-dimethoxymandelic acid for 28 days were observed by NaDCC treatment (Fig. 7). Considering that hexadecanoic acid, 9,12-octadecadienoic acid, oleic acid and octadecanoic acid are common fatty acids in plant membrane lipids while the contents of unsaturated fatty acids are involved in plant defense [48]. High concentrations of 9,12-octadecadienoic acid and oleic acid could enhance the pathogen resistance in the NaDCC-treated banana fruit during early storage, which was beneficial for delaying fruit ripening and senescence.

In comparison with organic acids, five alcohols were identified in the profiling of primary metabolites. Compared with the control fruit, NaDCC treatment increased the content of 2,3-butanediol, inositol, β-sitosterol and 9,19-cyclolanostan-3-ol by the end of the experiment. A previous study reported that stigmasterol is a significant indicator in bacterial infected leaf and is synthesized from β-sitosterol [49]. In this study, the decrease of stigmasterol could imply that NaDCC treatment promoted the resistance to bacterial infection. Additionally, inositol could be a precursor for the biosynthesis of plant cell walls [50].

Some other kinds of metabolites were found in the profiling of primary metabolites. After NaDCC treatment, the contents of acetamide, *N*-methyl-2-(2-hydroxyphenyl)ethylamine, 3,5-dimethoxymandelic amide, 1*H*-indole-3-ethanamine, 4-imidazolidinone, 2-pyrrolidinethione, benzeneethanamine, cadaverine and 3,4-dimethoxyphenylacetone decreased while ethylenediamine increased during storage (Fig. 7). However, their functions in relation to fruit ripening need to be investigated further.

## Methods

### Plant materials and treatments

Green mature fruit of banana (*Musa* spp., AAA group, cv. ‘Brazil’) were harvested from a commercial orchard in Guangzhou. Fruit with uniformity of shape, color and size were washed in water and then divided randomly into two groups. Based on the preliminary small-scale experiment (Additional file 1: Figure S1), NaDCC at 200 mg L^−1^ was chosen in this study. Fruit were submerged in a bath with 0 (water, control) and 200 mg L^−1^ NaDCC for 5 min at room temperature. After the treatments, the fruit were packed into plastic polyethylene bags (0.03 mm in thickness) and then stored at 25 ± 2 °C and 75–95% relative humidity (RH). Fruit from each treatment were randomly taken to measure color, fruit firmness, respiration rate and ethylene release rate. Mixed peel tissues from each treatment were collected, frozen in liquid nitrogen and then stored at − 20 °C and − 80 °C for physiological parameter analysis and RNA extraction, respectively.

### Determination of fruit color

Determination of skin color was measured with the Monolta chroma meter (CRC200; Minolta Camera Co., Tokyo, Japan). According to the method described by Huang et al. [13], five fruit fingers from each treatment were measured the peel color. For each fruit finger, three equidistant points around the middle of the fruit surface were determined with the chroma meter. Color was recorded using CIE L*, a* and b*. L* indicates the lightness or darkness and a* means green to red color while b* denotes blue to yellow color. Hue angle (h^0^) was calculated using the formula h^0^ = tan^−1^(b*/a*).

### Determination of fruit firmness

Banana fruit firmness was measured with a penetrometer (Model GY-3, Zhejiang Scientific Instruments, Zhejiang, China) according to the method of Huang et al. [13]. Five fruits were measured while each fruit finger was detected at three equidistant points around the middle position with the flat probe. Fruit firmness was expressed in Newton (N).

### Determinations of respiration and ethylene release rates

According to the method of Huang et al. [13], respiration rate was measured using an infrared gas analyzer (Li-6262 CO_2_/H_2_O analyzer, LI-COR, Inc, USA). Before being put into a plastic container (2.4 L) at 25 °C, three replicates of nine fruits from each treatment were weighted. The amount of CO_2_ was recorded for 5 min. The respiration rate was expressed as nmol kg^−1^ s^−1^.

Ethylene release rate was analyzed by the method of Huang et al. [13]. Three fruits were weighted and then placed into a 2.4 L plastic container. After 2 h, 10 mL of the headspace volume was sampled into a glass container, and then a sample (1 mL) was injected into the gas chromatography (GC-2010; Shimadzu, Kyoto, Japan) equipped with a 30 m HP-PLOT Q capillary column (Agilent Technologies, USA) and a flame ionization detector to measure the amount of ethylene production. Ethylene release rate was expressed as mmol kg^−1^s^−1^.

### Assays of 2,2-diphenyl-1-picrylhydrazyl (DPPH) radical scavenging activity and reducing power

Peel tissues (2.0 g) were ground and extracted with 20 mL methanol for 30 min. The extractions were centrifuged at 15,000×*g* for 20 min at 25 °C and then the supernatants was collected for analyses of DPPH radical scavenging activity and reducing power according to the method of Huang et al. [51].

The DPPH radical scavenging activity was evaluated by mixing 0.1 mL of the above-mentioned supernatant with 2.9 mL of 0.1 mM DPPH dissolved in methanol solution and then the absorbance was measured at 517 nm and three replicates were determined. The DPPH radical scavenging activity (%) of the sample was calculated by the method of Huang et al. [13].

The reducing power was measured by mixing 0.1 mL of the above-mentioned supernatant with 2.5 mL of 0.2 mM phosphate buffer (pH 6.6) and 2.5 mL of 1% potassium ferricyanide and then incubated for 20 min at 50 °C. Then 2.5 mL of 10% trichloroacetic acid was added and placed for 10 min. Finally, 5 mL of distilled water and 1 mL of 0.1% ferric chloride were added. The absorbance was measured at 700 nm and three replicates were determined.

### Measurement of hydroxyl radical scavenging activity

Hydroxyl radical scavenging activity was measured by the method described by Huang et al. [13] with some modifications. Frozen peel tissues (1.0 g) were crushed into powder and extracted with 10 mL methanol. The extraction solution was incubated for 30 min at 25 °C using ultrasonic treatment. The supernatant was collected after centrifuge at 15,000×*g* for 20 min at 25 °C. The reaction mixture containing 0.1 mL of the supernatant and 1 mL of reaction buffer (100 μM ferric chloride, 104 μM EDTA, 2.5 mM H_2_O_2_, 2.5 mM desoxyribose and 100 μM l-ascorbic acid) was incubated for 1 h at 37 °C, then mixed with 1 mL of 0.5% thiobarbituric acid dissolved in 0.025 M NaOH and 1 mL of 2.8% trichloroacetic acid and finally incubated for 30 min at 80 °C. After the mixture cooled down to 25 °C, the absorbance was measured at 532 nm. The reaction buffer was used as a blank. The hydroxyl radical scavenging activity was calculated by the method of Huang et al. [13].

### RNA isolation and real-time quantitative PCR (RT-qPCR) of genes

RNA was isolated according to the method of Jing et al. [52]. After grinding into powder, 10 g peel tissue were put into a 50 mL centrifuge tube, and then infunde 20 mL 80 °C preheated extracting buffer (0.2 M sodium borate, 30 mM EGTA, 1% sodium deoxycholate, 1% SDS, 10 mM DTT, 1%NP-40, 2% PVP-40) and 100 μL protease K. The extracts were put on the homogenizer for 2 h and infunded 2.4 mL 2 M potassium chloride then put into 4 °C freezer for 1.5 h. The extracts were then homogenized and centrifuge at 20,000 rpm for 30 min at 4 °C. Collecting the supernatants and add 1/3 of its origin volume 8 M lithium chloride then put into 4 °C freezer for 12–16 h. The extracts were centrifuged at 10,000 rpm for 30 min at 4 °C then outwelled the supernatants immediately and add 4 mL 2 M lithium chloride to wash the precipitates. After centrifuging and washing for 3 times, 4 mL 10 mM Tris–HCl (pH 7.5) were added into the precipitates. Until the precipitates dissolved entirely, 400 μL 2 M potassium acetate were added into the tube, and then the extracts were put into 4 °C freezer for 30 min. The extracts were centrifuged at 10,000 rpm for 15 min at 4 °C, then transferring the supernatants into new 15 mL centrifuge tubes. 10 mL 100% ethyl alcohol were added into the extracts then put into − 80 °C freezer for 2 h. The extracts were centrifuge at 10,000 rpm for 30 min at 4 °C, outwelled the supernatants and added 5 mL 70% ethyl alcohol. After washing the precipitates, the extracts were centrifuged at 10,000 rpm for 15 min at 4 °C then outwell the liquid. The precipitates were dried for 30 min under vacuum condition. After this step, the dry RNA were dissolved with 200 μL ddH_2_O and then transferred into a new 1.5 mL centrifuge tubes. The RNA samples were stored in the − 80 °C freezer. The total RNA was cleaned with DNase (TaKaRa Bio, Inc., Otsu, Shiga, Japan) and then DNA-free RNA was reverse transcribed using a PrimeScript^RT^ Master Mix reverse transcriptase Kit (TaKaRa: DRR036A).

RT-qPCR was conducted according to the method of Li et al. [53] using a 7500 fast real-time PCR system (Applied Biosystems, Foster City, CA, USA). The relative levels of gene expression were calculated according to the 2^−ΔΔCT^ method with *MAActin7* gene as the reference gene. The specific primers for *MaEBF1*, *MaERF1B*, *MaACO*, *MaACS*, *MaPECS*-*1.1* and *MaXTH9* are shown in Table 1. Three independent biological replicates were conducted.

**Table 1:** Specific primer sequences used in this study

Gene	Query name	Primer sequences (5′-3′)	Description
*MaACS*	Ma01_t07800.1	F:CGCCGTTGCCAATGACATCCAC R:GAGGTACTGCGTCTGCGAAGAGAT	1-Aminocyclopropane-1-carboxylate synthase CMA101
*MaACO*	GSMUA_AchrUn_randomT20420_001	F:GCACCAAGGTGAGCCACTAT R:TGGAAGAGGAGGATGACACC	1-Aminocyclopropane-1-carboxylate oxidase
*MaERF1B*	GSMUA_Achr11T22020_001	F:ACAAGAAAGCAAAGGAGAGTGAGACGAG R:CAGCAAGTGTTGGCTACTTCTGATGTTC	Ethylene-responsive transcription factor 1B
*MaEBF1*	GSMUA_Achr9T28510_001	F:AGTTGCTCTGTGCTTGATGACCTTGAT R:GGCAGACTCTTCAGTGTAACCTGTGAG	Putative EIN3-binding F-box protein1
*MaPECS*-*1*	GSMUA_Achr11T05430_001	F:ATATAAAGGCGGGGGCATAC R:CAGACCTGAAGGTGGTCCAT	Pectinesterase 3
*MaXTH9*	GSMUA_Achr6T19100_001	F: GAGGTGGATGGCGAATGGTA R: TCTGCAGTGACCTTGCCGTA	Xyloglucan endotransglucosylase/hydrolase
*MaACT7*	GSMUA_Achr11T12570_001	F:TGGTATGGAAGCCGCTGGTA R:TCTGCTGGAATGTGCTGAGG	Actin-7

### GC–MS analysis of primary metabolites

Primary metabolomics analysis was conducted by the method of Zhu et al. [38] with minor modifications. Sample (200 mg) was added to the extraction solution containing 1.800 mL methanol while 200 μL of 0.2 mg mL^−1^ ribitol dissolved in water was used as a quantification internal standard. The extraction solution was incubated for 15 min at 4 °C using ultrasonic treatments and then held for 15 min at 70 °C. After putting into a − 20 °C freezer for 0.5 h, the extraction was centrifuged for 15 min at 5000×*g* and 4 °C. Then, 100 μL of the supernatant was collected for the derivatization reaction. The derivation reaction was incubated in 80 μL of 20 mg mL^−1^ methoxyamine hydrochloride in pyridine for 1.5 h at 37 °C and then 80 μL of *N*-methyl-*N*-(trimethylsilyl) trifluoroacetamide (MSTFA) was added and placed for 0.5 h at 37 °C. The obtained sample (1 μL) was injected for GC–MS analysis (GC–MS-QP2010 Plus, Shimadzu Corporation, Kyoto, Japan) with the DB-5MS stationary phase fused-silica capillary column (30 m × 0.25 mm i.d., 0.25 μm, Agilent Technologies Inc., California, USA). The flow rate of carrier gas (99.999% helium) flow rate was 1.2 mL min^−1^. The column temperature was kept at 100 °C for 1 min, then increased to 184 °C at a rate of 3 °C min^−1^ and 190 °C at 0.5 °C min^−1^ and held for 1 min, and finally increased to 280 °C at 15 °C min^−1^ and held for 5 min. The ionization voltage of the MS was 70 eV and the interface temperature was 250 °C. The spilt ratio was 10:1 and the TIC (total ion current) spectra was scanned at a range from 45 to 600 m/z.

Datas presented in this study were the mean values of three replicates. The compounds were identified and accepted by searching in GC–MS analytical laboratories (NIST05 database) and some references of related studies. After normalization analysis according to the total peak area, the relative qualification of these compounds was based on the peak area ratio of quotation ions of the internal standard.

### Statistical analysis

The results of the experiments were expressed as the mean values of three biological replicates. The significant differences of the results were determined by the independent-sample T-test (*p *< 0.05) using SPSS version 16.0.

## Conclusion

This study showed that NaDCC treatment delayed ripening process and extended storage time of harvested banana fruit. The NaDCC treatment inhibited ethylene production and respiration rates and increased the antioxidant ability. Furthermore, the treatment inhibited the expressions of ethylene synthesis-related and cell wall degradation-related genes. Additionally, NaDCC treatment enhanced of the accumulation of some primary metabolites possibly involved in pathogen resistance.

Overall, application of NaDCC provided a potential postharvest treatment for extending shelf life during storage and transportation of banana fruit.

## Additional files

**Additional file 1: Figure S1.** Visual appearance of the banana fruit in small-scale experiment. (A): Control; (B): 50 mg L^−1^ NaDCC; (C): 100 mg L^−1^ NaDCC; (D): 200 mg L^−1^ NaDCC.

**Additional file 2: Figure S2** GC-MS profiles of the primary metabolites from banana peels. 27: Ribitol which was used as internal standard.

**Additional file 3: Table S1.** Detailed information of the identified primary metabolites.

## Abbreviations

NaDCC: sodium dichloroisocyanurate
DPPH: 2,2-diphenyl-1-picrylhydrazyl
ACO: 1-aminocyclopropane-1-carboxylate oxidase
ACS: 1-aminocyclopropane-1-carboxylate synthase
ERF: ethylene-responsive transcription factor
EBF: EIN3-binding F-box protein
PECS: pectinesterase
XTH: xyloglucan endotransglucosylase/hydrolase
ACT: actin
