# Supplementary material for: Sodium dichloroisocyanurate delays ripening and senescence of banana fruit during storage
Source: Chem Cent J. 2018 Dec 5;12:131. doi: 10.1186/s13065-018-0503-5 (PMC6768313; doi:10.1186/s13065-018-0503-5)
Supplement: Supplementary file 1 — Additional file 1: Figure S1. Visual appearance of the banana fruit in small-scale experiment. (A): Control; (B): 50 mg L−1 NaDCC; (C): 100 mg L−1 NaDCC; (D): 200 mg L−1 NaDCC. [file 13065_2018_503_MOESM1_ESM.docx]

**Figure. S1.** Visual appearance of the banana fruit in small-scale experiment. (A): 0 mg L^-1^ NaDCC (control); (B): 50 mg L^-1^ NaDCC; (C): 100 mg L^-1^ NaDCC; (D): 200 mg L^-1^ NaDCC.

**
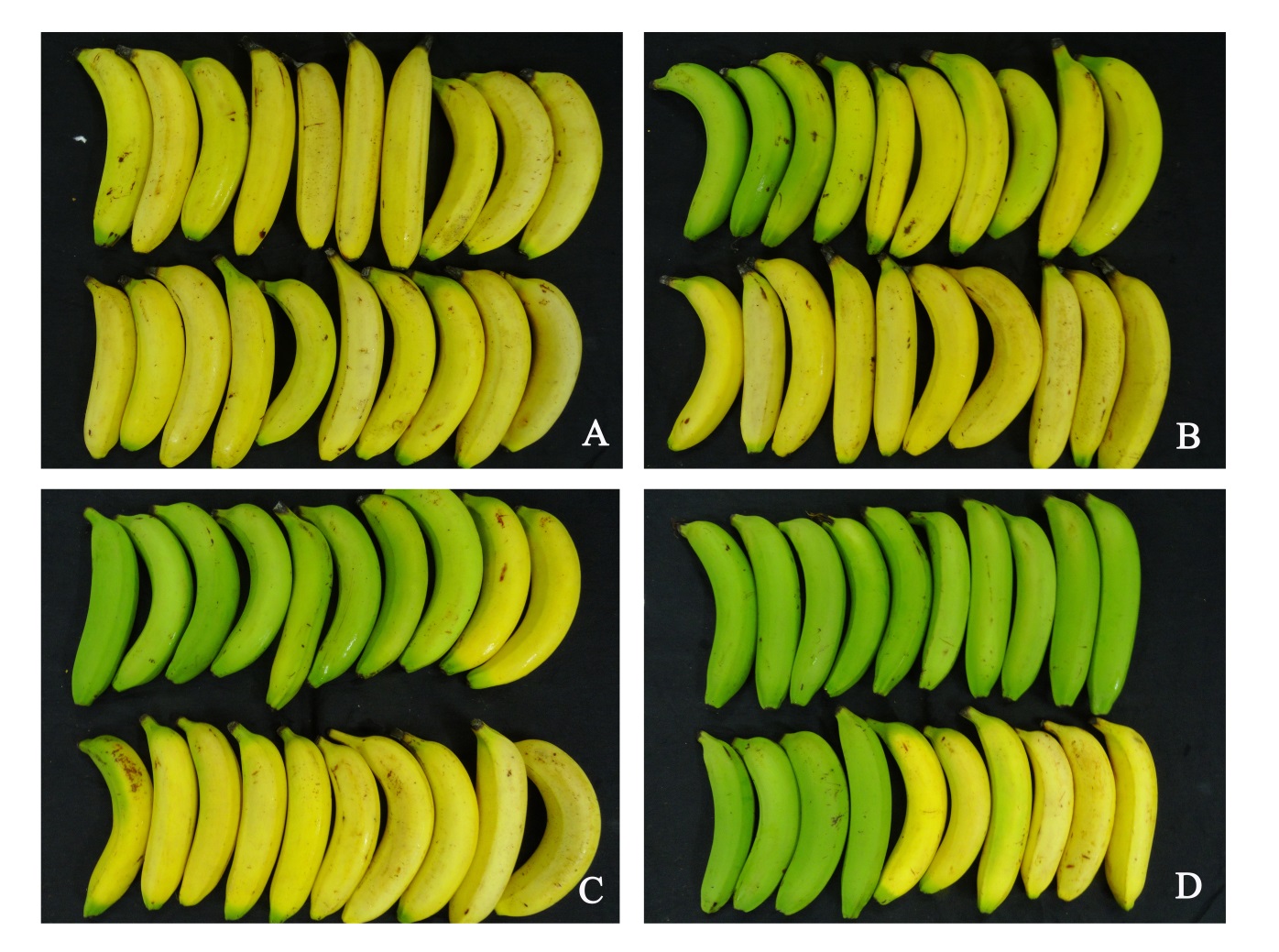
**

**Figure. S1**
