# Supplementary material for: Sodium dichloroisocyanurate delays ripening and senescence of banana fruit during storage
Source: Chem Cent J. 2018 Dec 5;12:131. doi: 10.1186/s13065-018-0503-5 (PMC6768313; doi:10.1186/s13065-018-0503-5)
Supplement: Supplementary file 2 — Additional file 2: Figure S2 GC-MS profiles of the primary metabolites from banana peels. 27: Ribitol which was used as internal standard. [file 13065_2018_503_MOESM2_ESM.docx]

**Figure. S2.** GC-MS profiles of the primary metabolites from banana peels.

27: Ribitol which was used as internal standard.

**
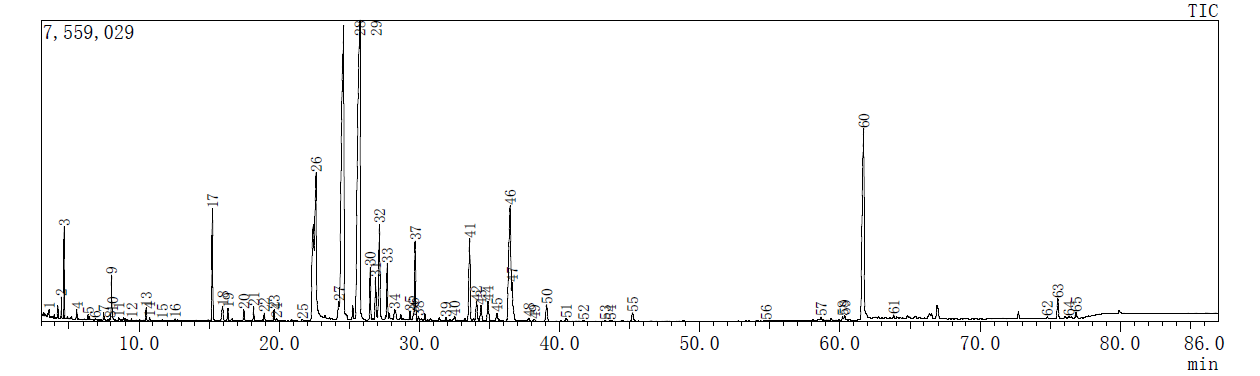
**

**Figure. S2**
