# Supplementary material for: Sodium dichloroisocyanurate delays ripening and senescence of banana fruit during storage
Source: Chem Cent J. 2018 Dec 5;12:131. doi: 10.1186/s13065-018-0503-5 (PMC6768313; doi:10.1186/s13065-018-0503-5)
Supplement: Supplementary file 3 — Additional file 3: Table S1. Detailed information of the identified primary metabolites. [file 13065_2018_503_MOESM3_ESM.docx]

**Table S1** Detailed information of the identified primary metabolites

| Number | Compound name | | Library similarity (%) | | Retention index | | Retention time | |
| --- | --- | --- | --- | --- | --- | --- | --- | --- |
| **Amino acids** | | | | | | | | |
| 6 | Glycylglycine  Serine  L-Norleucine  L-Threonine  L-Homoserine  L-Proline  L-Alanine  Glutamine  L-Asparagine  Glycine  L-Valine  L-Aspartic acid | | | 75 | | 1669 | | 6.864 |
| 7 |  |  |  | 78 | | 1158 | | 7.476 |
| 8 |  |  |  | 71 | | 1336 | | 7.924 |
| 11 |  |  |  | 90 | | 1193 | | 8.551 |
| 15 |  |  |  | 74 | | 1421 | | 11.651 |
| 18 |  |  |  | 94 | | 1466 | | 15.956 |
| 23 |  |  |  | 87 | | 1038 | | 19.615 |
| 24 |  |  |  | 83 | | 1612 | | 19.807 |
| 25 |  |  |  | 91 | | 1745 | | 21.627 |
| 35 |  |  |  | 84 | | 1061 | | 29.317 |
| 36 |  |  |  | 80 | | 1172 | | 29.565 |
| 45 |  |  |  | 75 | | 1512 | | 35.503 |
| **Organic acid** | | | | | | | | |
| 1 | Propanoic acid  Ethanedioic acid  Acetic acid  Malic acid  Butanoic acid  2-Keto-d-gluconic acid  Acetoxyacetic acid  3,4-Dimethoxymandelic acid  3,5-Dimethoxymandelic acid  2,5-Dimethoxymandelic acid  Hexadecanoic acid  9,12-Octadecadienoic acid | | | 92 | | 915 | | 3.576 |
| 3 |  |  |  | 97 | | 971 | | 4.66 |
| 10 |  |  |  | 80 | | 880 | | 8.094 |
| 17 |  |  |  | 93 | | 1390 | | 15.213 |
| 19 |  |  |  | 72 | | 1260 | | 16.324 |
| 31 |  |  |  | 85 | | 2073 | | 26.851 |
| 38 |  |  |  | 76 | | 1977 | | 29.919 |
| 39 |  |  |  | 78 | | 1867 | | 31.885 |
| 40 |  |  |  | 78 | | 1867 | | 32.494 |
| 42 |  |  |  | 83 | | 1867 | | 34.054 |
| 43 |  |  |  | 91 | | 1987 | | 34.375 |
| 53 |  |  |  | 79 | | 2202 | | 43.233 |
| 54 | Oleic acid | | | 83 | | 2194 | | 43.595 |
| 55 | Heptadecanoic acid | | | 80 | | 2087 | | 45.196 |
| 61 | Octadecanoic acid | | | 93 | | 2780 | | 63.83 |
| 63 | Cholan-24-oic acid | | | 74 | | 2922 | | 75.537 |
| **Sugars** | | | | | | | | |
| 14 | Mannose  D-Erythro-Pentopyranose  Sorbopyranose  D-Fructose  Glucopyranose  D-Glucose  β-D-Galactopyranoside  α-D-Glucopyranoside | | | 77 | | 1686 | | 10.731 |
| 21 |  |  |  | 75 | | 1446 | | 18.156 |
| 32 |  |  |  | 84 | | 2049 | | 27.161 |
| 34 |  |  |  | 88 | | 1982 | | 28.218 |
| 37 |  |  |  | 95 | | 2037 | | 29.717 |
| 41 |  |  |  | 96 | | 1970 | | 33.618 |
| 51 |  |  |  | 74 | | 1991 | | 40.395 |
| 60 |  |  |  | 94 | | 3552 | | 61.605 |
| **Alcohol** | | | | | | | | |
| 16 | 2,3-Butanediol  Inositol  Stigmasterol  β-Sitosterol  9,19-Cyclolanostan-3-ol | | | 76 | | 941 | | 12.57 |
| 49 |  |  |  | 84 | | 2194 | | 38.196 |
| 62 |  |  |  | 91 | | 2797 | | 74.784 |
| 64 |  |  |  | 83 | | 2789 | | 76.335 |
| 65 |  |  |  | 83 | | 2834 | | 76.834 |
| **Others** | | | | | | | | |
| 5 | Acetamide  N-Methyl-2-(2-hydroxyphenyl)ethylamine  3,5-Dimethoxymandelic amide  1H-Indole-3-ethanamine  4-Imidazolidinone  2-Pyrrolidinethione  Benzeneethanamine  Ethylenediamine  1,4-Butanediamine  Cadaverine  3,4-Dimethoxyphenylacetone | | | 74 | | 563 | | 6.341 |
| 22 |  |  |  | 87 | | 1548 | | 18.911 |
| 26 |  |  |  | 78 | | 1734 | | 22.738 |
| 28 |  |  |  | 86 | | 1839 | | 24.522 |
| 33 |  |  |  | 80 | | 1683 | | 27.687 |
| 46 |  |  |  | 70 | | 1613 | | 36.357 |
| 47 |  |  |  | 86 | | 2174 | | 36.583 |
| 48 |  |  |  | 79 | | 1557 | | 37.773 |
| 52 |  |  |  | 82 | | 1350 | | 41.68 |
| 56 |  |  |  | 83 | | 1449 | | 54.685 |
| 59 |  |  |  | 79 | | 1507 | | 60.32 |
| **Impurities** |  | | |  | |  | |  |
| 4 | Pentasiloxane | | | 96 | | 0 | | 4.448 |
| 9 | Silanol | | | 94 | | 1068 | | 5.56 |
| 12 | Trisiloxane | | | 93 | | 0 | | 8.034 |
| 13 | Asparagine | | | 85 | | 698 | | 9.456 |
| 20 | Silane | | | 70 | | 1754 | | 10.496 |
| 29 | Phenylpropanolamine | | | 91 | | 1498 | | 17.467 |
| 30 | 1-Methyl-5-mercaptotetrazole | | | 90 | | 1555 | | 25.771 |
| 44 | Silanamine | | | 83 | | 0 | | 26.49 |
| 50 | Tyrosine | | | 78 | | 1394 | | 34.876 |
| 57 | 1,3-Dipalmitin | | | 63 | | 2087 | | 39.067 |
| 58 | Dimethyl-(isopropyl)-silyloxybenzene | | | 76 | | 4055 | | 58.067 |
| **Internal standard** | |  | |  | |  | |  |
| 27 | Ribitol | | | 88 | | 1746 | | 24.258 |
